# Supplementary material for: Chromatin conformation analysis of primary patient tissue using a low input Hi-C method
Source: Nat Commun. 2018 Nov 29;9:4938. doi: 10.1038/s41467-018-06961-0 (PMC6265268; doi:10.1038/s41467-018-06961-0)
Supplement: Supplementary file 1 — Supplementary Information [file 41467_2018_6961_MOESM1_ESM.pdf]

Supplementary Information

Chromatin conformation analysis of primary patient tissue using a low input Hi-C method

Díaz and Kruse et al.

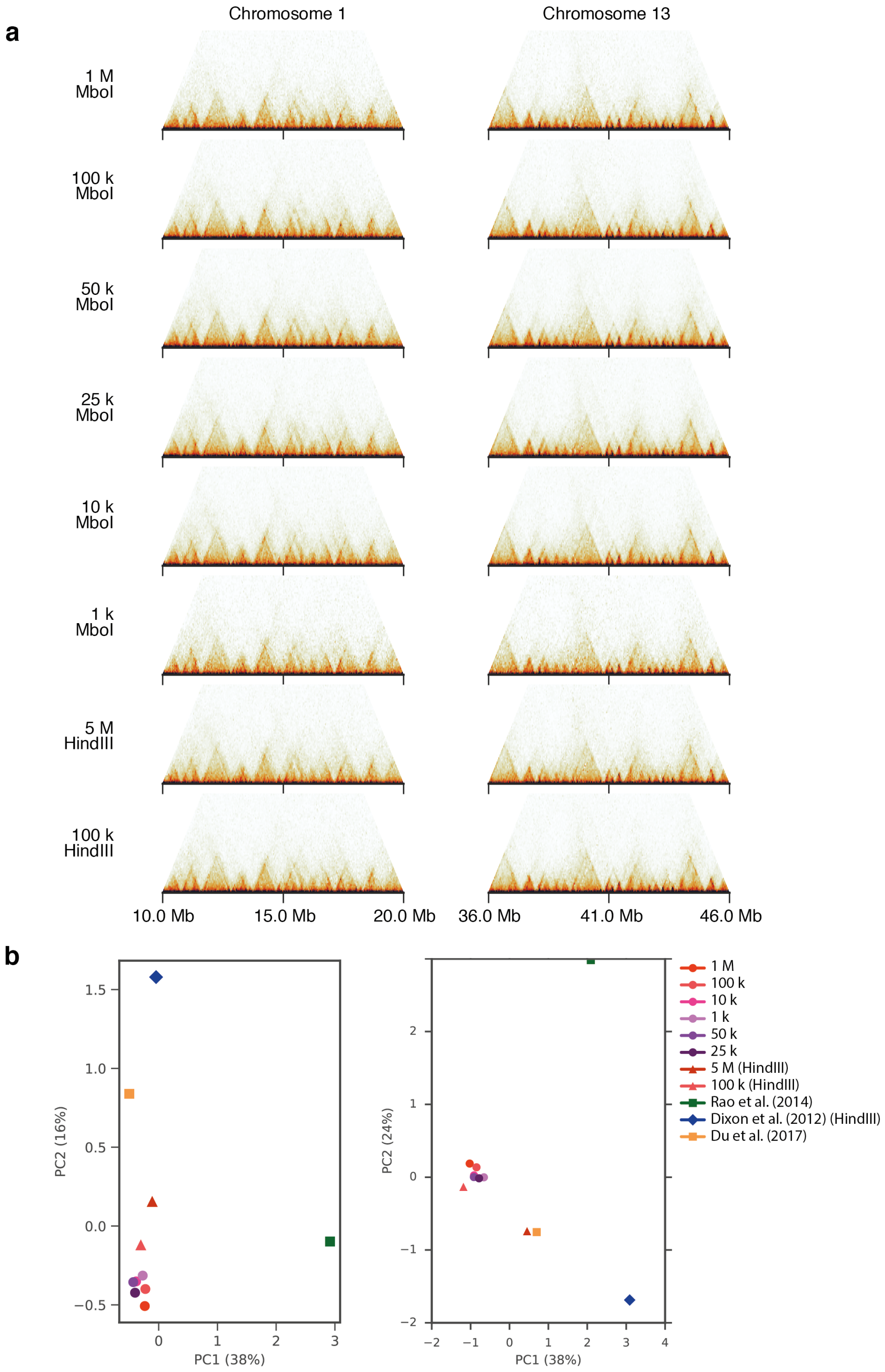

Supplementary Figure 1. Low-C results are highly similar for different input cell numbers. (a) Visual comparison of Hi-C matrices at 50 kb resolution from all mESC Low-C libraries in this study. Two different genomic regions are shown as examples. Input cell numbers are reported to the left of each matrix. (b) PCA on the top 50,000 most variable entries in the 100 kb resolution Hi-C matrices of contacts between 200 kb and 2 Mb (left) and 50 kb and 1 Mb (right) for all mESC Low-C samples. The plot also includes the mESC Hi-C datasets published by Dixon *et al.* (2012)[1], Du *et al.* (2017)[2], and the mouse CH12-LX dataset from Rao *et al.* (2014)[3]. Circles denote MboI, triangles denote HindIII in the Low-C samples. Dixon *et al.* used HindIII, Rao *et al.* and Du *et al.* used MboI.

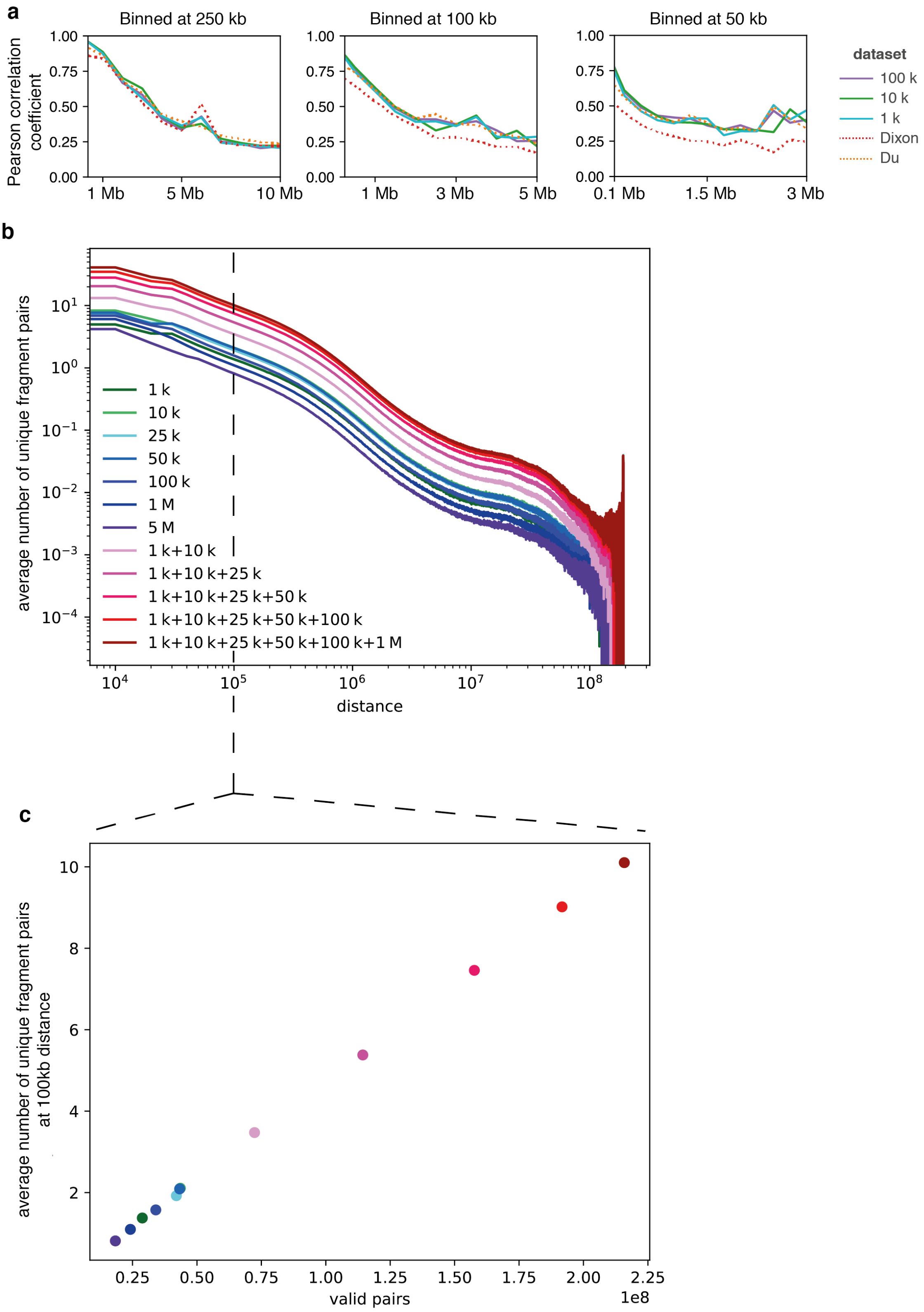

Supplementary Figure 2. Low-C library complexity is not affected by input cell number. (a) Same plot as Fig. 2a, but all datasets have been downsampled to the number of valid pairs in the 1 M Low-C library for comparison. (b) Line plot of the average number of unique fragment pairs observed at increasing fragment distances for all Low-C libraries and, additionally, merged Low-C libraries. (c) Average number of unique fragment pairs at 100 kb distance as a function of the number of valid fragment pairs in each library.

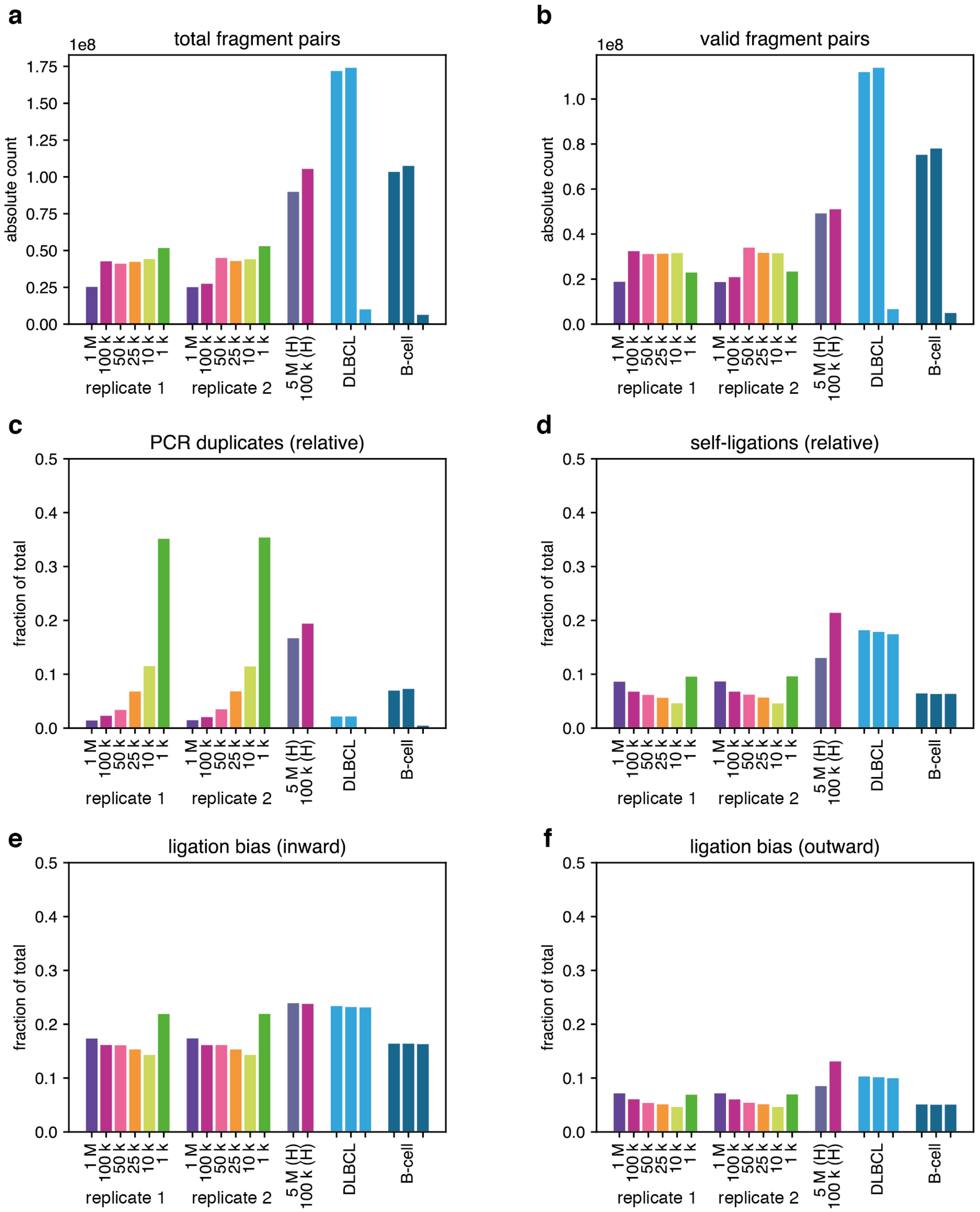

Supplementary Figure 3. Low-C library statistics**.** (a-b) Low-C fragment pair statistics (a) Total number of fragment pairs in each library. (b) Total number of valid fragment pairs after filtering for biases. (c-f) Different types of biases (see Methods) for each Low-C library expressed in fraction of total pairs. (c) PCR duplicates. (d) Self-ligated fragments. (e) Inward ligation error. (f) Outward ligation error. H=HindIII.

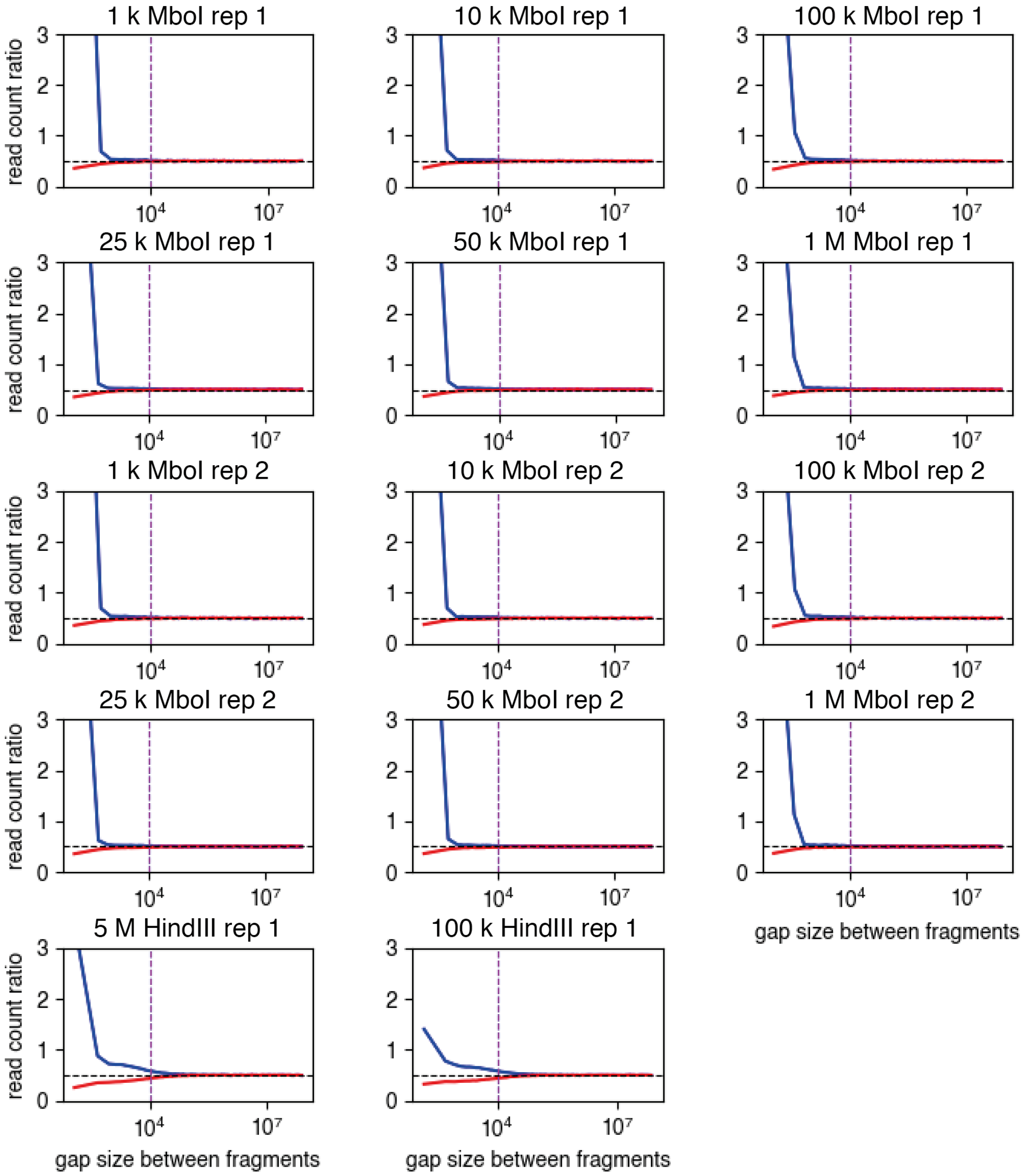

Supplementary Figure 4. Ligation error plots of Low-C samples using differing amounts of input material. Shown are ratios of paired reads facing towards each other (red; “inward”-facing, i.e. the first read is on the + strand and the second on the – strand) or away from each other (blue; “outward”-facing, i.e. the first mapped read is on the - strand, the second mapped read is on the + strand) to reads facing in the same direction (-/- or +/+)[4,5]. Vertical dotted lines represent cutoffs for filtering read pairs at distances < 10 kb.

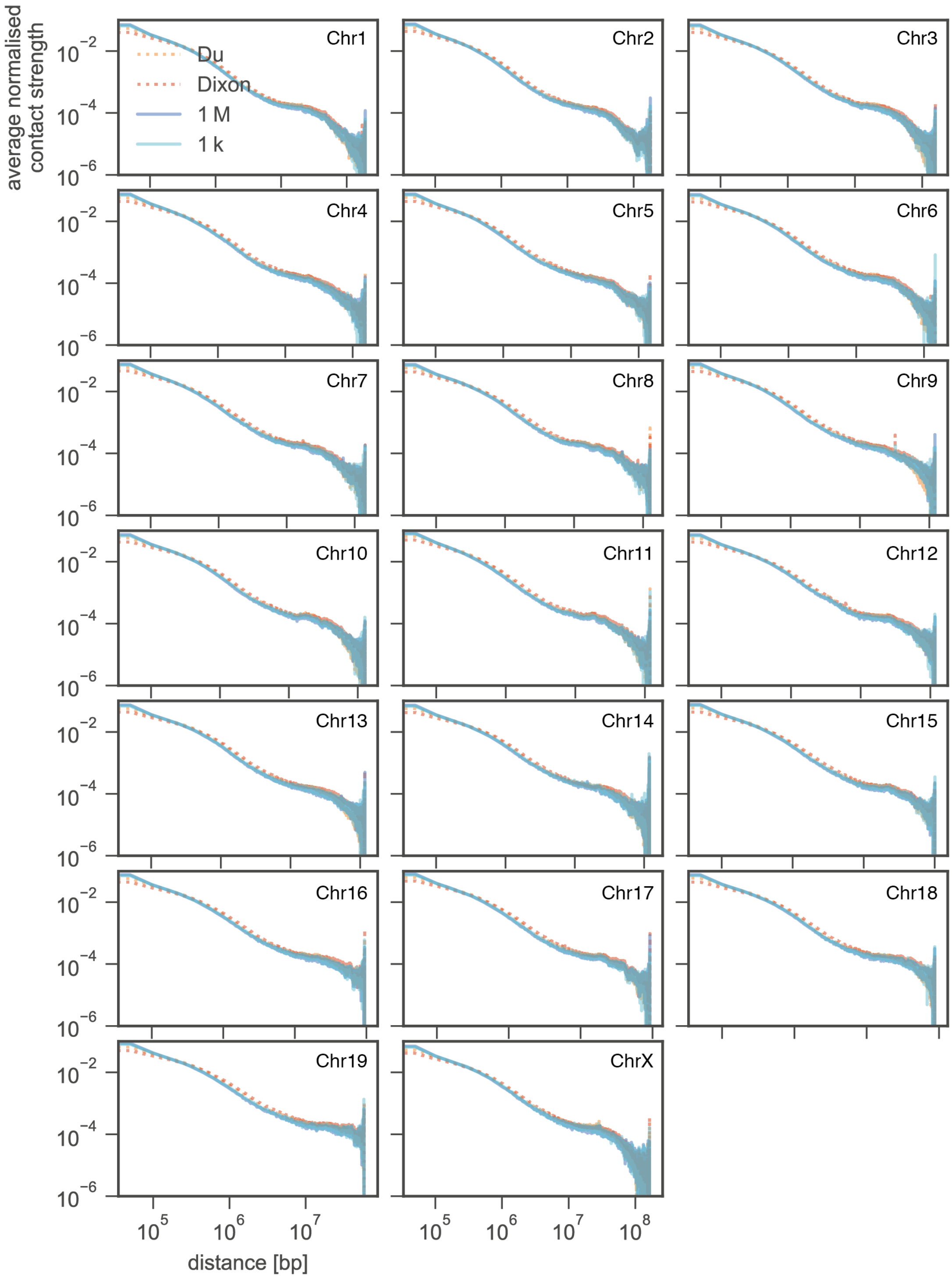

Supplementary Figure 5. Contact probability decay with distance (log-log plots) for each chromosome in the 1 k and 1 M samples, as well as Dixon *et al*. (2012)[1] and Du *et al.* (2017)[2] mESC Hi-C maps.

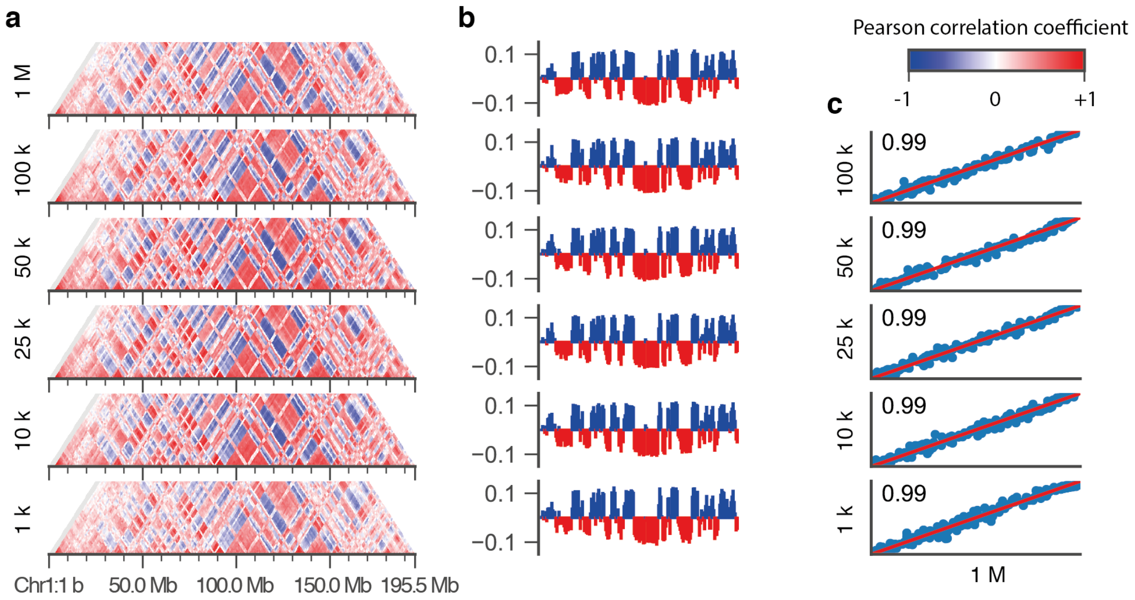

Supplementary Figure 6. AB compartment reproducibility for each Low-C mESC MboI sample. (a) Correlation matrices for chromosome 1. (b) First eigenvector of the matrices in (a), positive values are coloured blue, negative values red. (c) Scatter plots of the eigenvector values in (b) against the eigenvector of the 1 M sample with Pearson correlation coefficient R indicated in the top left corner. Red line indicates identity.

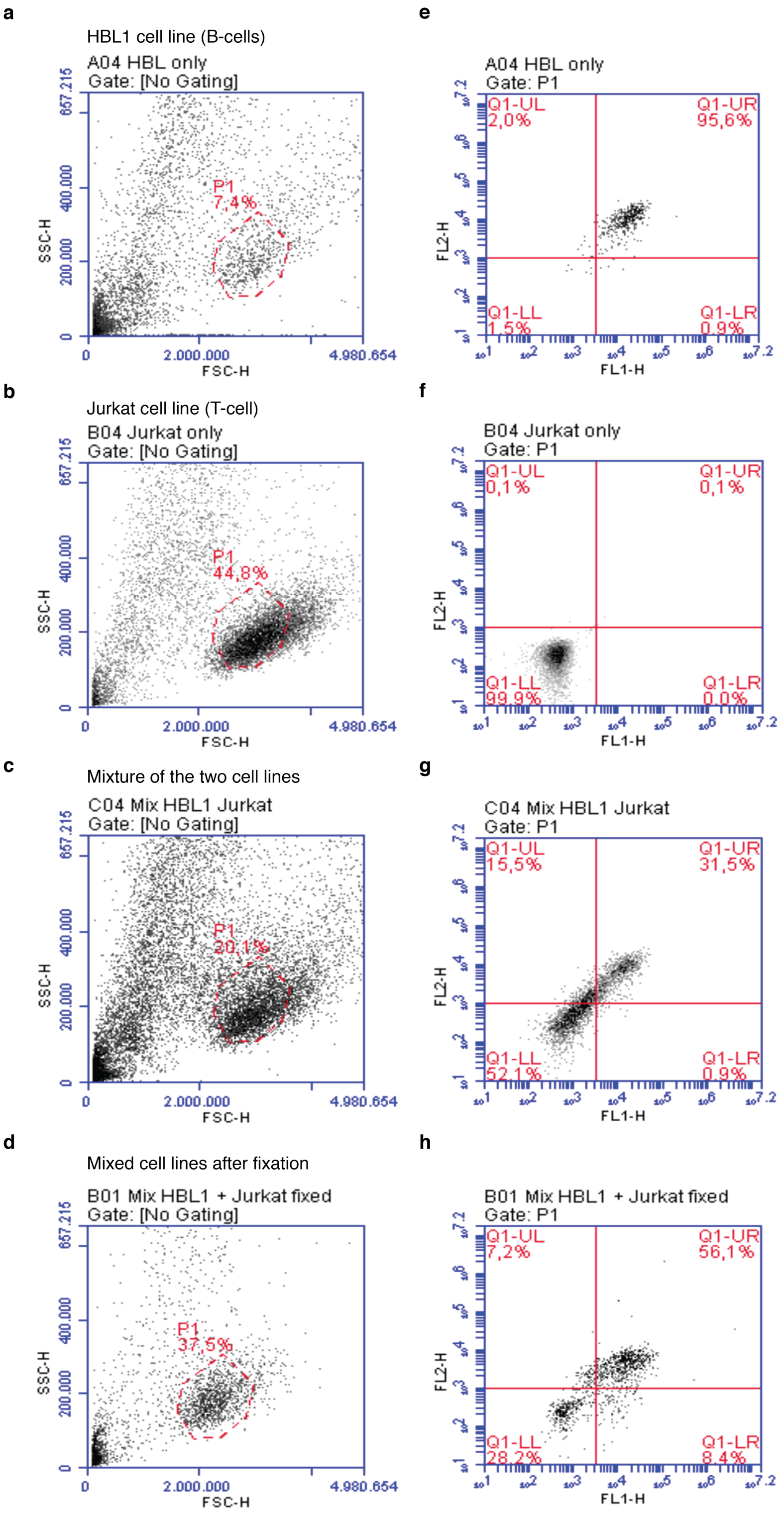

Supplementary Figure 7. Flow cytometry analysis for HBL1 and Jurkat cells. (a-d) Scatter density plots for a HBL1 (B-cell) cell line (a), Jurkat (T-cell) cell line (b), and a mixed HBL1-Jurkat cell population before (c) and after (d) formaldehyde fixation. Discontinuous red circles demarcate the cell population of interest. (e-h) Scatter plots for double-staining with CD20 (channel one; FL1-H) and CD19 (channel two; FL2-H) antibodies for HBL1 (e), Jurkat (f) and a mixed HBL1-Jurkat cell populations before (g) and after (h) formaldehyde fixation. Red lines demarcate the quadrants for positive/negative cell labelling.

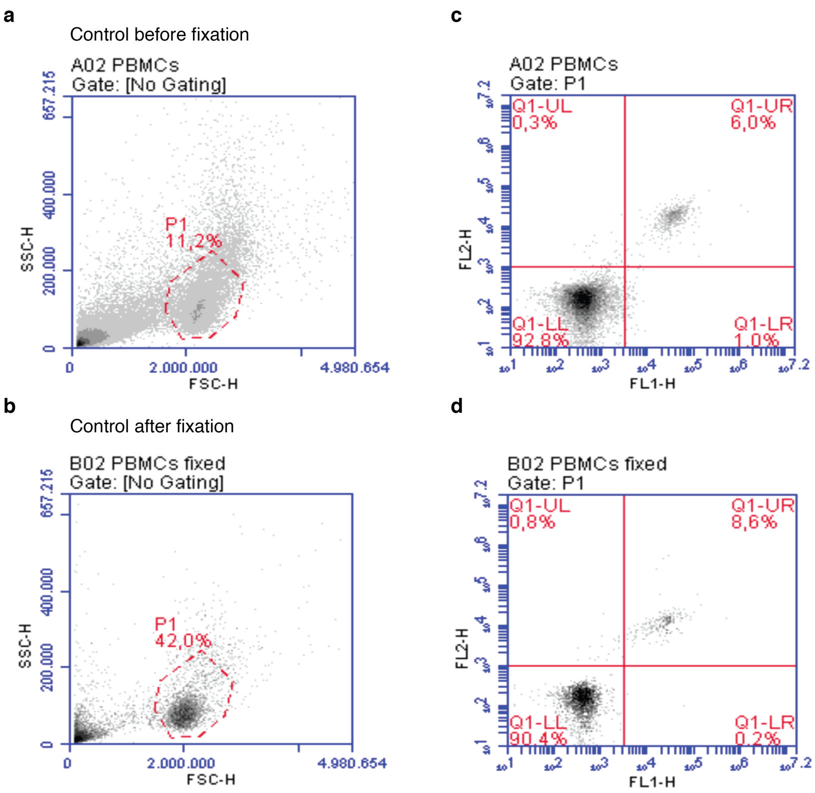

Supplementary Figure 8. Flow cytometry analysis of PBMCs. (a, b) Scatter density plots for the PBMC population before (a) and after (b) cell fixation with formaldehyde. Among PBMCs, lymphocytes are shown enclosed on a red discontinuous circle. (c, d) Scatter plots for double-staining of PBMCs with CD20 (channel one; FL1-H) and CD19 (channel two; FL2-H) antibodies, before (c) and after (d) formaldehyde fixation. Red lines demarcate the quadrants for positive/negative cell labelling.

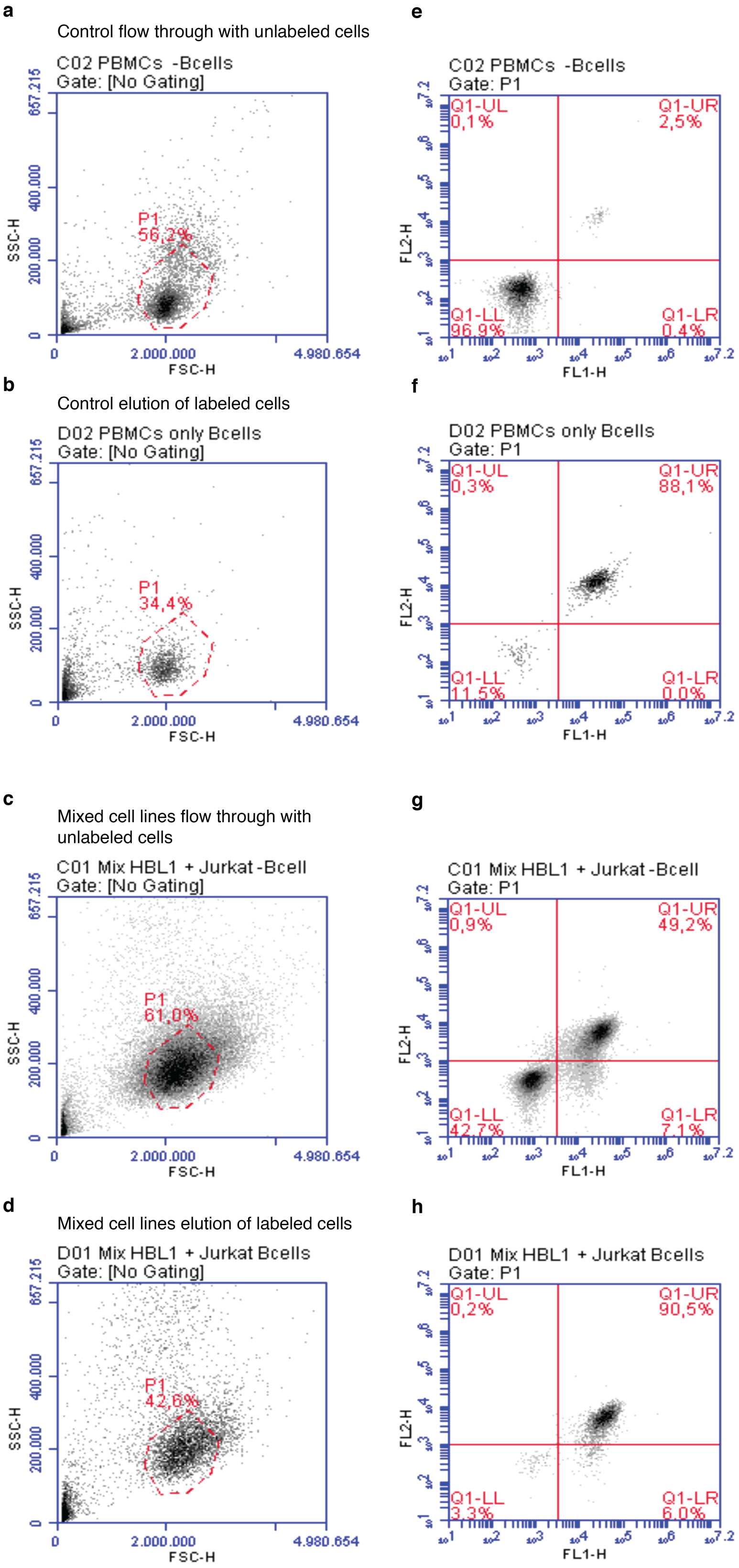

Supplementary Figure 9. Flow cytometry analysis on the MACS sorted populations from a healthy donor's PBMCs and a mixture of B- and T-cell population. (a-d) Scatter density plots for the healthy donor PBMCs without B-cells (a, MACS unlabelled CD20^{-} flow through), the healthy donor B-cells (b, MACS elution of labelled CD20^{+} cells), Jurkat (T-cells) from a mixed HBL1-Jurkat mixed cell population (c, MACS unlabelled CD20^{-} flow through), and HBL1 (B-cells) from the same HBL1-Jurkat mixed population (d, MACS elution of labelled CD20^{+} cells). Discontinuous red circles demarcate the cell population of interest. (e-h) Scatter-plots for double-staining of each cell population with CD20 (channel one; FL1-H) and CD19 (channel two; FL2-H) antibodies. Scatter plot for the healthy donor PBMCs without B-cells (e, MACS unlabelled CD20^{-} flow through), the healthy donor B-cells (f, MACS elution of labelled CD20^{+} cells), Jurkat (T-cells) from a mixed HBL1-Jurkat mixed cell population (g, MACS unlabelled CD20^{-} flow through), and HBL1 (B-cells) from the same HBL1-Jurkat mixed population (h, MACS elution of labelled CD20- cells). Red lines demarcate the quadrants for positive/negative cell labelling.

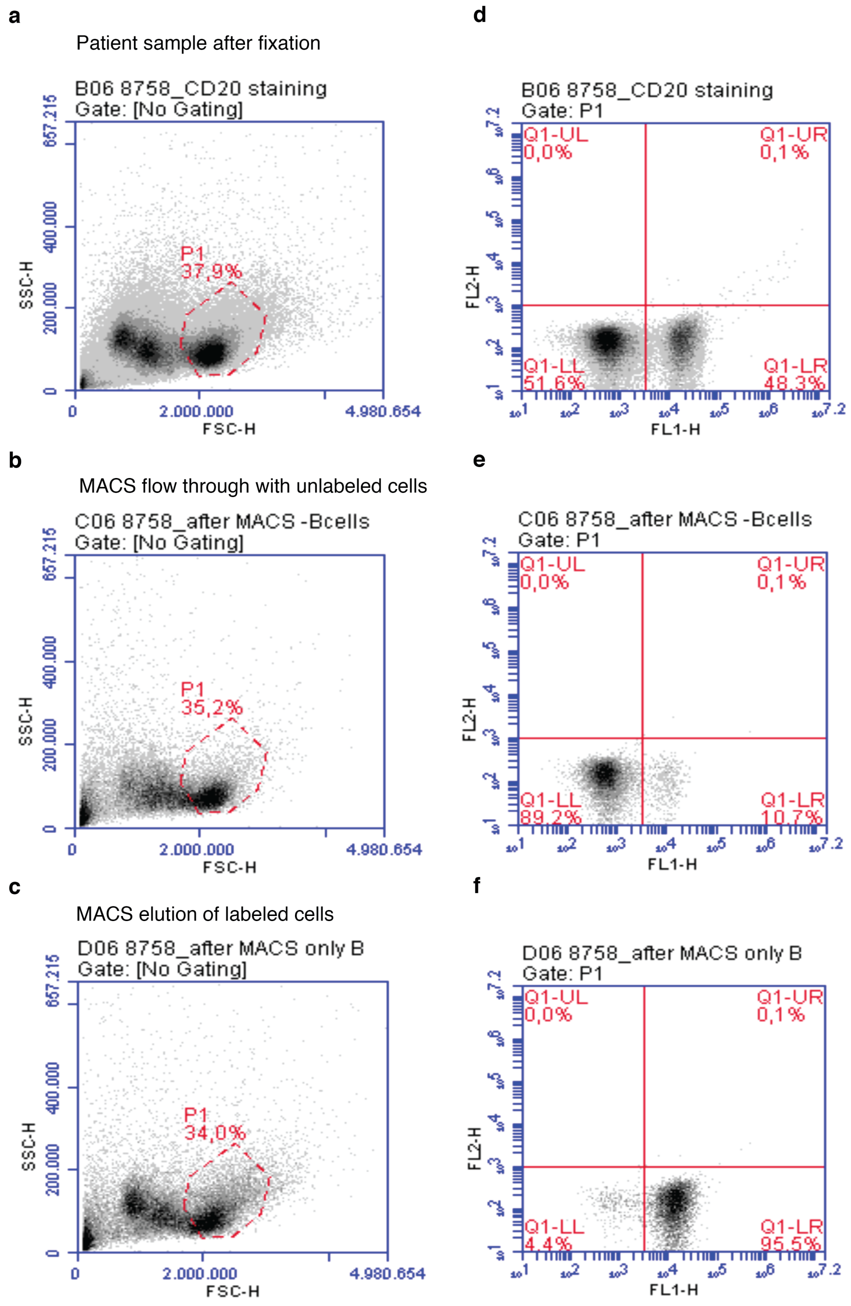

Supplementary Figure 10. Flow cytometry analysis of the patient sample after fixation with formaldehyde and MACS sorting. (a-c) Scatter density plots for the patient lymphocytes after formaldehyde fixation (a), patient MACS sorted lymphocytes without B-cells (b, MACS unlabelled CD20- flow through), and patient B-cells (c, MACS elution of labelled $CD20^+$ cells). Lymphocytes are shown enclosed in a red discontinuous circle. (d-f) Scatter-plots for double-staining of each cell population with CD20 (channel one; FL1-H) and CD19 (channel two; FL2-H) antibodies, for the patient lymphocytes after formaldehyde fixation (d), patient MACS sorted lymphocytes without B-cells (e, MACS unlabelled $CD20^-$ flow through), and patient B-cells (f, MACS elution of labelled $CD20^+$ cells). Red lines demarcate the quadrants for positive/negative cell labelling.

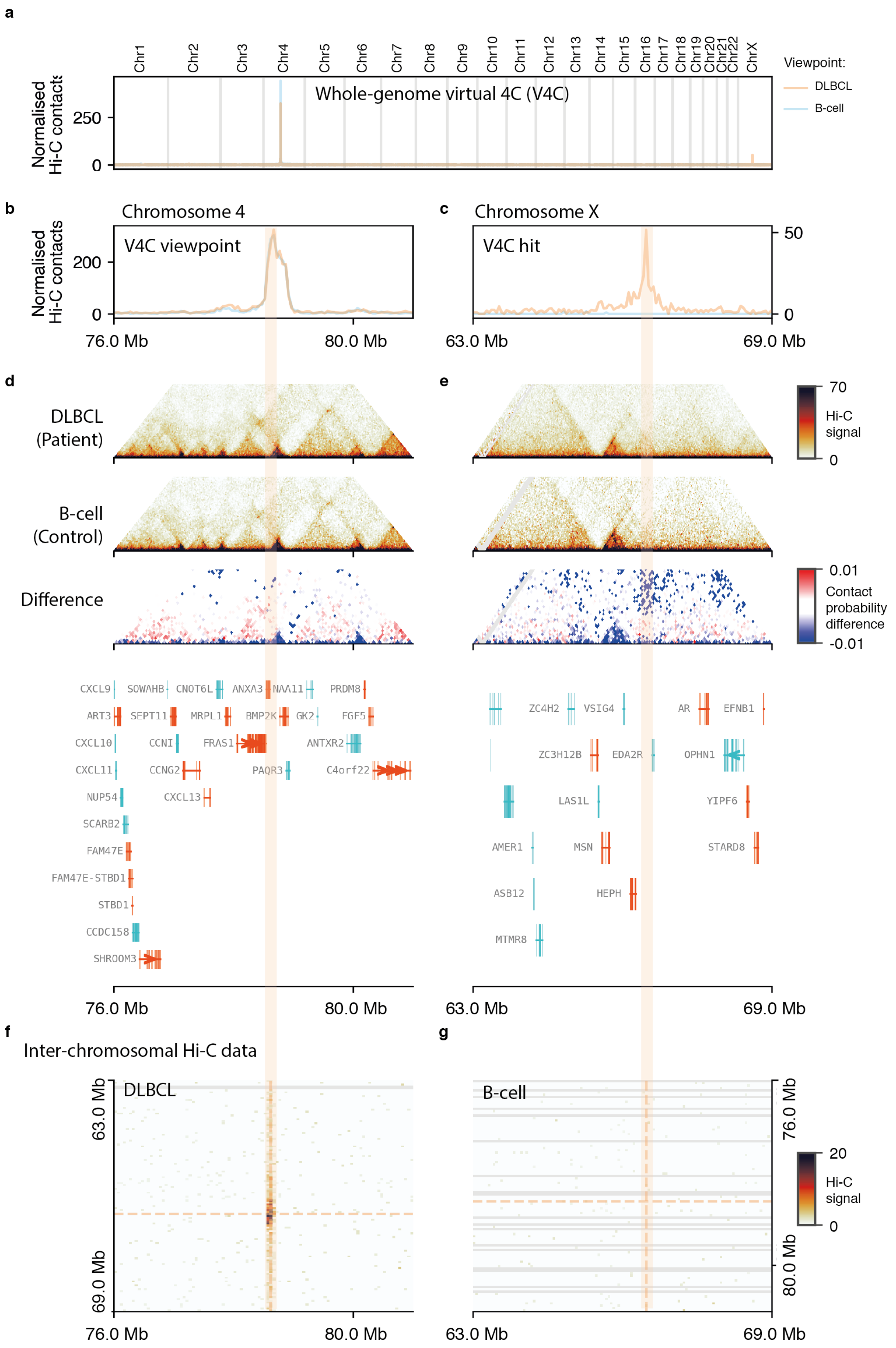

Supplementary Figure 11. Unbiased detection and characterisation of structural rearrangement at *ANXA3* in the DLBCL sample. (a) Whole-genome virtual 4C plot for two viewpoints on chromosome 4 (orange, control in blue). (b, c) Zoom-in of the virtual 4C plots to the viewpoint (b) and target (c) regions. (d, e) Local Hi-C maps of the viewpoint (d) and target (e) regions. (f, g) Inter-chromosomal Hi-C maps of target *vs.* viewpoint region in the patient (f) and viewpoint *vs.* target region in control (g).

Supplementary Table 1. Overview of Low-C samples in this study and their respective numbers of read pairs.

Sample	*Technical replicate*	*Restriction enzyme**	*Read pairs*	*Mapped read pairs*	*Valid read pairs (after filtering)*	*Total valid pairs (per condition)*
mouse						
1 M	1	M	45,373,559	25,251,575	18,798,179	37,499,845
1 M	2	M	45,521,764	25,140,413	18,701,666	
100 k	1	M	82,904,353	42,649,971	32,408,508	53,300,749
100 k	2	M	53,221,423	27,414,542	20,892,241	
50 k	1	M	60,840,152	44,930,010	33,976,593	65,130,281
50 k	2	M	66,595,079	41,112,150	31,153,688	
25 k	1	M	62,322,544	42,866,712	31,680,705	62,953,595
25 k	2	M	63,250,992	42,279,423	31,272,890	
10 k	1	M	65,602,065	44,080,826	31,497,073	63,027,306
10 k	2	M	65,489,378	44,080,826	31,497,073	
1 k	1	M	86,616,647	51,722,407	22,937,947	46,290,053
1 k	2	M	89,231,005	52,922,902	23,352,106	
5 M	1	H	123,453,028	89,807,393	49,189,133	49,189,133
100 k	1	H	140,373,791	105,378,368	51,030,522	51,030,522
human						
DLBCL	1	M	250,881,396	171,870,902	111,898,247	
DLBCL	2	M	252,231,324	174,080,064	113,844,746	232,446,650
DLBCL	3	M	14,289,185	10,030,808	6,703,657	
B-Cell	1	M	150,626,741	103,327,894	75,175,780	
B-Cell	2	M	155,140,165	107,468,078	77,957,359	158,093,470
B-Cell	3	M	9,001,104	6,340,367	4,960,331	

**M = MboI, H = HindIII*

Supplementary Table 2. Numbers of valid read pairs in *cis*/*trans* as a measure of quality control. Fraction *cis* in the last column is corrected for the relative amount of theoretically possible *trans* compared to *cis* contacts in each genome.

Sample	*Restriction enzyme*	*Cis*	*Trans*	*Fraction cis*	*Cis/trans*	*Fraction cis (corrected for genome size – see Methods)*
5 M	HindIII	42,641,237	6,500,291	0.868	6.560	0.991
100 k	HindIII	43,690,020	7,297,432	0.857	5.987	0.991
1 M	MboI	30,513,663	6,949,072	0.815	4.391	0.987
100 k	MboI	43,711,297	9,549,094	0.821	4.578	0.988
50 k	MboI	55,375,829	9,698,299	0.851	5.710	0.990
25 k	MboI	52,464,537	10,439,922	0.834	5.025	0.989
10 k	MboI	54,891,458	8,080,917	0.872	6.793	0.992
1 k	MboI	39,768,358	6,476,292	0.860	6.141	0.991
DLBCL	MboI	142,651,132	89,401,751	0.615	1.596	0.966
B-cell	MboI	98,490,584	59,345,608	0.624	1.660	0.967

Supplementary References

1. Dixon, J. R. *et al.* Topological domains in mammalian genomes identified by analysis of chromatin interactions. *Nature* **485,** 376–80 (2012).
2. Du, Z. *et al.* Allelic reprogramming of 3D chromatin architecture during early mammalian development. *Nature* **547,** 232–235 (2017).
3. Rao, S. S. P. P. *et al.* A 3D map of the human genome at kilobase resolution reveals principles of chromatin looping. *Cell* **159,** 1665–1680 (2014).
4. Cournac, A., Marie-Nelly, H., Marbouty, M., Koszul, R. & Mozziconacci, J. Normalization of a chromosomal contact map. *BMC Genomics* **13,** 436 (2012).
5. Jin, F. *et al.* A high-resolution map of the three-dimensional chromatin interactome in human cells. *Nature* **503,** 290–4 (2013).
