# Supplementary material for: Chromatin conformation analysis of primary patient tissue using a low input Hi-C method
Source: Nat Commun. 2018 Nov 29;9:4938. doi: 10.1038/s41467-018-06961-0 (PMC6265268; doi:10.1038/s41467-018-06961-0)
Supplement: Supplementary file 3 — Description of Additional Supplementary Files [file 41467_2018_6961_MOESM3_ESM.pdf]

Description of Additional Supplementary Files

File Name: Supplementary Data 1
Description: (Excel file: Supplementary-Data-1-protocol.xlsx) Supplementary data legend: Step-by-step Low-C experimental protocol.

File Name: Supplementary Data 2
Description: (Excel file: Supplementary-Data-2-regionchanges.xlsx) Supplementary data legend: List of genomic regions and genes ranked by magnitude of conformational differences between the DLBCL and B-cell Hi-C maps.
